# Interpretation of SD-OCT imaging data in real-life conditions versus standardized reading centre analysis in eyes with diabetic macular oedema or macular oedema secondary to retinal vein occlusion: 24-month follow-up of the ORCA study

**DOI:** 10.1007/s00417-024-06579-7

**Published:** 2024-09-19

**Authors:** Georg Spital, Steffen Schmitz-Valckenberg, Bettina Müller, Erika Liczenczias, Petrus Chang, Britta Heimes-Bussmann, Focke Ziemssen, Sandra Liakopoulos, For the ORCA study group

**Affiliations:** 1 M3 Reading Centre, Eye Centre at the St. Franziskus Hospital, Münster, Germany; 2 GRADE Reading Centre, Department of Ophthalmology, University of Bonn, Bonn, Germany; 3 Department of Ophthalmology & Visual Sciences, John A. Moran Eye Centre, Salt Lake City, UT USA; 4 Novartis Pharma GmbH, Nuremberg, Germany; 5 Centre for Ophthalmology, Eberhard Karls University, Tübingen, Germany; 6 Department of Ophthalmology, Leipzig University Hospital, University of Leipzig, Leipzig, Germany; 7 Cologne Image Reading Centre, Department of Ophthalmology, Faculty of Medicine and University Hospital Cologne, Cologne, Germany; 8 Department of Ophthalmology, Goethe-University, Frankfurt, Germany

**Keywords:** Diabetic macular oedema (DME), Macular oedema (ME), Retinal vein occlusion (RVO), Anti-VEGF, Spectral Domain Optical coherence tomography (SD-OCT)

## Abstract

**Purpose:**

As part of the prospective, non-interventional OCEAN study, the ORCA module evaluated physicians’ spectral domain optical coherence tomography (SD-OCT) image interpretations in the treatment of diabetic macular oedema (DME) or macular oedema (ME) secondary to retinal vein occlusion (RVO).

**Methods:**

Presence of intraretinal fluid (IRF) and/or subretinal fluid (SRF) was evaluated independently by physicians and reading centres (RCs) on 1612 SD-OCT scans of 133 patients diagnosed with either DME or ME secondary to RVO. Agreement between physicians and RCs was calculated for both cohorts individually and as a combined ME cohort. Physicians’ treatment decisions were analysed related to the results of the OCT-evaluations.

**Results:**

For the combined ME cohort, presence of IRF/SRF was recorded by RCs in 792/1612 (49.1%) visits and by physicians in 852/1612 (52.9%) visits, with an agreement regarding presence or absence of foveal fluid in 70.4% of cases. In 64.4% (510/792) of visits with RC-detected foveal IRF and/or SRF no injection was given. In 30.3% of these visits with foveal fluid no reason was identified for a ‘watch and wait’ approach indicating possible undertreatment. BCVA deterioration was seen in a quarter of these eyes at the following visit.

**Conclusion:**

Despite good agreement between physicians and RCs to recognize SRF and IRF, our data indicate that omitting injections despite foveal involvement of fluid is frequent in routine clinical practice. This may put patients at risk of undertreatment, which may negatively impact real-life BCVA outcomes.

**Trial registration:**

www.clinicaltrials.gov, identifier NCT02194803.

**Supplementary Information:**

The online version contains supplementary material available at 10.1007/s00417-024-06579-7.

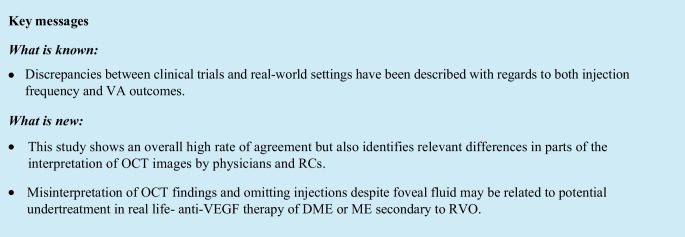



## Introduction

In both diabetic retinopathy (DR) and retinal vein occlusion (RVO), macular oedema (ME) may disrupt the retinal morphology and impair vision [1]. Intravitreal anti-vascular endothelial growth factor (anti-VEGF) therapeutics improve retinal morphology and function [2–4] and are a first-line treatment option for both conditions [5, 6]. Retreatment criteria rely on a combination of changes in visual acuity (VA) and monitoring of the extent, localisation, and temporal duration of retinal fluid accumulation visible on optical coherence tomography (OCT). Regular control visits and adequate interpretation of OCT scans are therefore essential to determine when retreatment is required [7].

In real-world clinical settings, treatment outcomes frequently do not match those reported in clinical trials, which may be at least partly due to misinterpretation of OCT imaging findings in addition to multiple other clinician- and patient-dependent factors [8–10]. Accurate interpretation of OCT findings is crucial for optimal treatment decisions.

The ORCA module of the 24-month OCEAN study compared the interpretation of spectral domain OCT (SD-OCT) scans by physicians and RCs in cohorts of patients undergoing anti-VEGF (ranibizumab) treatment for neovascular age-related macular degeneration (nAMD) [8], diabetic macular oedema (DME) or ME secondary to RVO [11–13]. The comparative analysis between the assessment of the initial diagnosis at baseline by treating physicians and Reading Centres (RCs) has already been reported [14].

In the current analysis, longitudinal data from ORCA patients diagnosed with either DME or ME secondary to RVO were assessed to determine the accuracy of OCT interpretations by treating physicians and the adequacy of physicians’ treatment decisions over 24 months of follow-up in real-world clinical practice.

## Methods

The cohorts analysed were comprised of patients from the ORCA module as part of the prospective, multi-centre, non-interventional Phase IV OCEAN study^1^ [8, 11, 13]. Physicians at clinical sites performing SD-OCT examinations as part of their routine clinical care could participate in the ORCA module [8]. To allow adequate evaluation of SD-OCT volume scans, the RCs developed a consensus for minimum quality requirements for colour fundus photography (FP), fluorescein angiography (FA) and SD-OCT imaging that were aligned with criteria issued by professional associations [15, 16]. A handbook for image acquisition and image data transmission to the RCs was provided to the participating sites.

Patients who had not received anti-VEGF injections within three months prior to study entry or any previous treatment with intravitreal steroids with RC-confirmed diagnosis of DME or ME secondary to RVO were included. Subjects were followed over 24 months at 36 ophthalmic centres in Germany. Information on best corrected visual acuity (BCVA) at all visits, number and type of anti-VEGF injections, and any additional treatments were documented.

Collected imaging data included all routinely performed SD-OCT scans (Optopol Copernicus, Zeiss, Topcon and Heidelberg Engineering) and available FP and FA. Images were evaluated by treating physicians through a standardised OCT questionnaire and by three independent RCs.^2^ All RCs were blinded to the results of the physicians [14].

Retinal thickness parameters, calculated from the internal limiting membrane to Bruch’s membrane, included central subfield retinal thickness (CSRT, i.e., the average thickness in the central 1 mm^2^ area of the fovea) and foveal centre point thickness (FCPT, i.e., the thickness at the fovea as measured on the most central B-scan). CSRT values were provided by the automated viewing software after checking for correct segmentations and a correctly centred positioning of the ETDRS grid. In case of segmentation errors, manual correction of segmentation lines was performed if the required time to perform the correction was less than 2 min.

OCT signs of disease activity were defined as presence of intraretinal fluid (IRF) and/or subretinal fluid (SRF) with foveal involvement and/or diffuse foveal thickening (Online Resource 1). In addition to the comparison of the OCT evaluation, the adequacy of the physicians’ treatment indications was assessed by the RCs based on OCT imaging findings, also considering the course of oedema and the course of VA. Possible reasons for suspected undertreatment or overtreatment were evaluated. Grading options for presence of IRF/SRF included ‘yes’, ‘no’, ‘questionable’ and ‘cannot grade’. For statistical analysis, ‘questionable’ and ‘cannot grade’ were counted as ‘no’, as no definite presence was confirmed. The RCs also evaluated whether an OCT parameter was strongly or discreetly visible. The statistical analyses were descriptive.

## Results

### Study population

A total of 147 patients were identified by physicians as having either DME (n = 88) or ME secondary to RVO (n = 59). RCs confirmed the diagnosis in 133/147 (90.5%) of these patients (DME 87.5% and RVO 94.9%). Patients excluded due to conflicting diagnoses are summarised in Online resource 2. From the patients with confirmed diagnoses, 1612 follow-up visits (DME 978, RVO 634) with SD-OCT scans evaluated by physicians and RCs were available for analysis. Patients had a mean age of around 70 years, 50.7% of DME and 64.3% of RVO patients were female. The majority (67.7%) was treatment naïve (Online Resource 3).

### Physician versus RC agreement on OCT signs of disease activity

Presence of IRF and/or SRF with foveal involvement was recorded in 792/1612 (49.1%) scans by RCs (DME: 56.2%, RVO: 38.2%) and in 852/1612 (52.9%) scans by physicians (DME 59.7%, RVO 42.3%). For 1054 scans SD-OCT evaluations were available from both RCs and physicians with agreement on presence of foveal fluid in 59.7% (629/1054) and agreement on absence or questionable presence of foveal fluid in 10.7% (113/1054) of all scans for the combined ME cohort. Foveal IRF/SRF detected by the RCs were correctly identified by the physicians in 85.2% (629/738 visits) and absence of foveal IRF/SRF as evaluated by the RCs was correctly identified in 35.8% (113/316 visits) (data for DME and RVO cohort in Table 1).

**Table 1:** Degree of consensus in the evaluation of fluid with foveal involvement in SD-OCT scans from follow-up visits in the ORCA-DME and ORCA-RVO cohorts

**ORCA-DME cohort:** **(** ***n=*** 707)*	**Reading Centre**	
	IRF and/or SRF present: *n=*510 (72.1%) [100%]	IRF and/or SRF absent or presence questionable: *n=*197 (27.9%) [100%]
**Physician**		
IRF and/or SRF present: *n=*572 (80.9%)	Agreement *n=*437 (61.8%) [85.7]	Discrepancy *n=*135 (19.1%) [68.2]
IRF and/or SRF absent or IRF/SRF presence questionable: *n=*135 (19.1%)	Discrepancy *n=*73 (10.3%) [14.3%]	Agreement *n=*62 (8.8%) [31.5]
**ORCA-RVO cohort:** **(** ***n=*** 347)*	**Reading Centre**	
	IRF/SRF present: *n=*228 (65.7%) [100%]	IRF/SRF absent or IRF/SRF presence questionable: *n=*119 (34.3%) [100%]
**Physician**		
IRF and/or SRF present: *n=*260 (74.9%)	Agreement *n=*192 (55.3%) [84.2%]	Discrepancy *n=*68 (19.6%) [57.1%]
IRF and/or SRF absent or IRF/SRF presence questionable: *n=*87 (25.1%)	Discrepancy *n=*36 (10.4%) [15.8%]	Agreement *n=*51 (14.7%) [42.9%]

*Only scans with available SD-OCT evaluations from both RCs as well as physicians are shown (DME 707/978, RVO 347/634)

Numbers in [square brackets] show percentage of RC evaluated scans

*DME*, diabetic macular oedema; *IR*F, intraretinal fluid; *RC*, reading centre; *RVO*, retinal vein occlusion; *SD-OCT*, spectral domain-optical coherence tomography; *SRF*, subretinal fluid

**Fig. 1:**
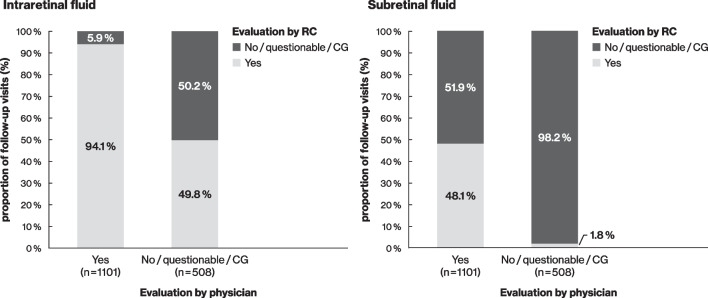
Agreement between physicians and RCs for the identification of IRF and SRF in complete SD-OCT volume scans of follow-up visits. Evaluations of the presence of IRF (on the left) and SRF (on the right) were compared. The percentages shown are calculated based on a 100% ‘Yes’ or 100% ‘No/questionable/cannot grade’ evaluation of the physician for each parameter. For some scans, information on the parameter was missing/not assessable by the physician or the RCs: intraretinal fluid, n = 8; subretinal fluid, n = 13. CG, cannot grade; NA, not assessable; IRF, intraretinal fluid; RC, reading centre; SD-OCT, spectral domain-optical coherence tomography; SRF, subretinal fluid

When looking at IRF and SRF in the whole OCT volume scan-field, RCs confirmed the fluid detection of physicians in 94.1% of scans for IRF (DME 96.0%, RVO 90.5%) and in 48.1% of scans for SRF (DME 40.6%, RVO 53.3%). Absence of IRF was confirmed in 50.2% (DME 36.1%, RVO 64.1%), and absence of SRF in 98.2% (DME 98.7%, RVO 97.3%) (Fig. 1). 49.8% (253) of scans (DME 63.9%, RVO 35.9%) with absence of IRF according to the physicians were considered false negative by the RCs. In 102 of these 253 scans (40.3%) [DME 82/161 (50.9%) and RVO 20/92 (21.7%)] RCs evaluated IRF to be strongly visible.

For SRF, false negative evaluations were documented by physicians in 1.8% (28) of scans where they estimated absence or questionable presence of fluid (43.1% of RC detected SRF). RCs evaluated SRF as strongly visible in 11 of these scans (39.3%).

The degree of change in IRF/SRF with foveal component relative to the previous visit reported by physicians and RCs is presented in Online Resource 4. Agreement between RCs and physicians was found in 61.9% of scans with physician-reported increase in IRF/SRF since the previous visit. In 78.8% of scans physicians reported stable IRF/SRF (defined as IRF and SRF not changing over three consecutive visits), this finding was confirmed by the RCs. In 53.6% of scans evaluated to show decreased IRF/SRF since the previous visit, the result was consistent to the RC evaluation. In 34.1% of scans, physicians reported increase of IRF/SRF whereas RCs reported some of them as stable (33.7%) or even decreased (4.5%). This disagreement was higher in DME (RC-reported stable: 37.2%; RC-reported decreased: 5.8%) than in RVO (RC-reported stable: 28.6%; RC-reported decreased: 2.5%).

### Physician versus RC agreement on retinal thickness measurements

Physicians and RCs considered the automatic segmentation of the retinal thickness in the foveal centre subfield to be adequate in 92.6% and 79.9% of scans, respectively (DME 92.9% and 84.3%; RVO 92.1% and 73.2%), with agreement on correct segmentation between physicians and RCs in 75.1% of scans. The agreement was higher in the DME cohort (80.2%) than in the RVO cohort (67.4%). In 4.7% of scans, physicians evaluated the segmentation as incorrect, compared to 18.7% by RCs evaluation.

The position of the EDTRS grid was manually corrected by physicians and RCs in 9.3% and 41.3% of scans, respectively (DME 8.7% and 35.6%; RVO 10.3% and 50.2%) (Online Resource 5).

CSRT measurements in the combined cohort were quantified by physicians and RCs to be 298.6 ± 28.2 µm and 302.2 ± 85.1 µm, respectively, while FCPT was 280.2 ± 93.9 µm and 265.3 ± 108.6 µm respectively. In the DME cohort, no significant difference was found for physician and RC evaluations of CSRT (physicians 305.7 ± 63.4 µm; RCs 307.3 ± 65.8 µm; mean difference: 0.3 ± 21.1 µm; *P* = 0.67; *n* = 930 scans). The evaluation of CSRT in the RVO cohort showed a significant but small difference (physicians 287.5 ± 104.0 µm; RCs: 294.4 ± 107.7 µm; mean difference: -4.5 ± 31.3 µm; *P* < 0.001; n = 599 scans). FCPT measurements differed significantly between physicians and RCs in both cohorts (Online Resource 6).

### VA outcomes and evaluation of possible over- or undertreatment

**Fig. 2:**
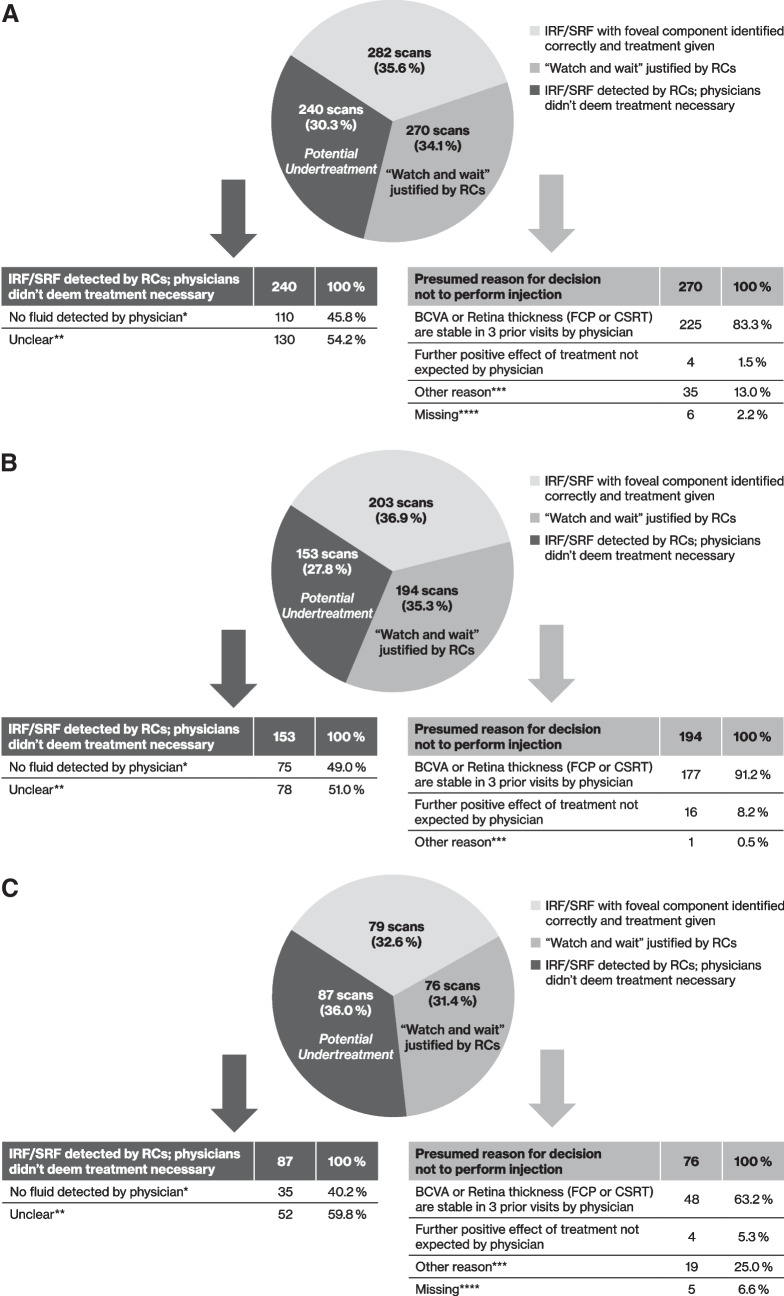
Identification of IRF/SRF parameters on SD-OCT scans and treatment decisions by physicians for 1612 scans with RC confirmed foveal IRF/SRF in the combined ME cohort (**A**); ORCA-DME cohort (**B**); ORCA-RVO cohort (**C**). Presumed reasons for decisions not to perform injection are presented. *In 79/110 fluid was considered clearly visible by RCs. **Intra- and/or subretinal fluid with foveal component was observed by the physician. ***Other reasons include reasons such as the injection was planned for a later visit, patient lost to follow-up, no further injections requested, therapy change, patient preferences, adverse events, and other reasons without further clarification. ****Missing information were classified under ‘Watch and wait’ justified to avoid over-estimating undertreatment. CSRT, Central subfield retinal thickness; DME, diabetic macular oedema; FCP, foveal centre point; IRF, intraretinal fluid; RC, reading centre; SD-OCT, spectral domain optical coherence tomography, SRF, subretinal fluid

Subsequent anti-VEGF injections were administered in 282 of 792 (35.6%) cases with RC diagnosis of fovea involving IRF/SRF (DME 203/550 [36.9%]; RVO 79/242 [32.6%]). Analysing the decisions not to treat despite fovea involving oedema in detail, the RCs judged a ‘watch and wait’ approach taken by the physicians as acceptable at 270/792 (34.1%) visits (194/550 [35.3%] for DME and 76/242 [31.4%] for RVO), mainly because BCVA or retina thickness were classified as stable in 3 prior visits by physicians (225/270 [83.8%]). For DME this was the case at 177/194 (91.2%) visits and for RVO at 48/76 (63.2%) visits. In 240/792 (30.3%, DME: 153/550 [27.8%], RVO: 87/242 [36.0%]) visits, physicians did not deem treatment necessary, however these cases could not be explained by stable BCVA or retinal thickness. The reasons for this decision were unclear (DME 78/153 [51.0%] visits; RVO 52/87 [59.8%] visits) or seemed to be the consequence of a false-negative evaluation of foveal fluid (DME 75/153 [49.0%] visits; RVO: 35/87 [40.2%] visits) (Fig. 2a-c).

Among the patients that were not injected albeit RCs detected signs of foveal IRF and/or SRF and no comprehensive reason for not giving an injection could be found, stable vision (± 5 ETDRS letters) for the following visit was reported for 63.9% (DME 62.3%; RVO 66.2%) of visits. Deterioration in BCVA in the following visit was seen in a quarter of cases (DME and RVO 27.3% and 24.7%, respectively). Gain of ≥ 15 ETDRS letters was recorded at 6.0% of combined ME cohort visits (DME 5.0%; RVO 7.8%) and loss of > 15 ETDRS letters was recorded at 3.7% of combined ME cohort visits (DME 2.2%; RVO 6.5%) (Online Resource 7).

Anti-VEGF injection was administered at 323/820 (39.4%) visits where presence of fovea involving oedema could not be confirmed by the RCs (DME 177/428 [41.4%] visits; RVO 146/392 [37.2%] visits). Injection could be justified in 153/323 (47.4%) of these visits (DME 83/177 [46.9%]; RVO 70/146 [47.9%]) as physicians reported a visual decline compared to the previous visit (69/153 visits; DME 35 visits; RVO 24 visits) or the injection was performed within a series (84/153 visits; DME 48 visits; RVO 36 visits). However, at 170/820 (23.7%) visits (DME 94/428 [22.0%]; RVO 76/428 [19.4%]) a possible overtreatment by physicians may be suspected as injections were given despite no obvious reason. Furthermore, in 46 of these 170 (27.1%) visits (DME 28/94 [29.8%]; RVO 18/76 [23.7%]) the physicians reported presence of IRF and/or SRF with foveal component although there was none according to RC evaluation. In 124/428 (29.0%) scans (DME 66 scans; RVO 58 scans) the reasons for injection remained unclear. Based on the RC evaluation, 77/133 (57.9%) patients (DME 48/77 [62.3%]; RVO 29/56 [51.8%]) were deemed by RCs to be possibly undertreated in at least one visit, and 66/133 (49.6%) patients (DME 37/77 [48.1%]; RVO 29/56 [51.8%]) were deemed to be possibly overtreated in at least one visit.

### Assessment of physicians’ level of experience and confidence at interpreting OCT images

The treating physicians generally reported high levels of confidence in their evaluations of the SD-OCT scans, stating in 95.5% of scans to be confident or very confident of their evaluations (Online Resource 8). Most physicians (70.4% 19/27) stated more than 2 years of experience in interpreting OCT scans, four physicians (14.8%) had 1–2 years, and 3 physicians (11.1%) 1–12 months of experience (Online Resource 9).

## Discussion

OCT-guided anti-VEGF therapy is an established first line treatment option for both DME and ME secondary to RVO [5, 6, 17, 18]. Discrepancies between clinical trials and real-world settings have been noted with regards to both injection frequency and VA outcomes [19–24].

This study shows an overall high rate of agreement but also identifies relevant differences in parts of the interpretation of OCT images by physicians and RCs. Misinterpretation of OCT images may be related to potential undertreatment or possible overtreatment. These results support the hypothesis that OCT scan misinterpretation contributes to unsatisfactory outcomes as previously reported for real-life situations [25, 26].

In contrast to data for nAMD in the ORCA module, the automatic OCT-segmentation was deemed correct for most cases of ME secondary to RVO or DME. Even if the data showed significant differences between the physicians and RCs, the localisation of the fovea and the correct positioning of the ETDRS grid seemed to be less challenging in DME and in ME secondary to RVO than in nAMD [14]. Identification of foveal centre and a correct grid position is mandatory for a correct measurement of CSRT and FCPT.

The levels of agreement on presence or absence of IRF and/or SRF with foveal involvement between physicians and RCs were higher for scans where physicians reported presence of IRF/SRF than for scans where physicians reported absence of IRF/SRF [27, 28].

Physicians reported false positive IRF/SRF approximately twice as frequently as false negative among both the DME and RVO cohort patients. Although false positive OCT interpretations may lead to overtreatment, it is unlikely to have a negative impact on VA outcomes but may incur avoidable costs to the healthcare system and increase the risk of treatment-related adverse events [8].

We assume that underestimation of IRF/SRF by physicians has contributed to the fact that in two thirds of visits with foveal oedema no injection was given. Although false-negative evaluation of IRF and/or SRF by physicians was low, this could be related to approximately one quarter of OCT scans that did not lead to anti-VEGF injection despite fovea involving oedema.

Other possible reasons for not injecting include stability of VA and/or stability of edema over three consecutive visits. However, in about 30% of all cases with foveal oedema no treatment was given and no comprehensive reason for a possible ‘watch and wait’ procedure could be discovered.

The number of no-treatment decisions in cases where foveal IRF/SRF was detected by RCs suggests that the interpretation of OCT scans may constitute an opportunity for improvement in real-life clinical practice decision making. Even if the negative effect of SRF and IRF on retinal function and integrity is much more pronounced and more rapid in nAMD compared to ME caused by retinal vascular diseases, a BCVA deterioration was seen in the following visit in a quarter of cases (DME and RVO 27.3% and 24.7%, respectively) with presumed undertreatment. That finding suggests that a more rigorous treatment regimen may have been beneficial for optimal preservation of vision over time in these patients. Real-life studies demonstrated that undertreatment often leads to unsatisfying results in treatment of DME and in ME secondary to RVO. To achieve an effective treatment frequency in an as needed anti-VEGF-therapy an adequate number and a correct interpretation of OCT-examinations apparently seems to be crucial.

Study limitations include the non-interventional design of the ORCA module, small cohort sizes; heterogeneity of German OCT systems, and proprietary data formats and regulatory requirements [29]. Additionally, since standard physician OCT image interpretation skills were required for study participation, more experienced physicians may be even over-represented, which may have led to underestimation of the true real-world extent of deviating assessments. Also, individual reasons for treatment discontinuation despite correctly detected signs of IRF/SRF were not regularly captured, which limits treatment discontinuation analysis.

In summary, the overall approximately 70% consensus rate for determining presence or absence of IRF/SRF between independent RCs and treating physicians indicates that IRF/SRF is often correctly identified in real-life clinical practice conditions. In contrast to nAMD, segmentation problems of the OCT algorithm are rare in retinal vascular diseases and the retinal thickness measurements of the CSRT and the FCPT seem to be robust parameters, likely due to the clear identification of retinal borders and the foveal centre. Misinterpretation of OCT images and omitting injections despite presence of foveal fluid may have contributed to potential undertreatment in up to two thirds (62.3%) of ORCA-DME patients and half of (51.8%) ORCA-RVO patients, which may negatively impact VA outcomes. Misinterpretation of OCT scans could also result in overtreatment in a subset of patients, thus increasing the risk of treatment-related side effects and costs to the healthcare system. In conclusion, improving treating physicians’ ability of adequate SD-OCT interpretation by education programs along with additional help in daily OCT evaluation by future artificial intelligence assistance, may lead to improved clinical outcomes and more precise anti-VEGF treatment for patients diagnosed with DME or ME secondary to RVO.

## Supplementary Information

Below is the link to the electronic supplementary material.

Supplementary file1 (PDF 488 KB)

## Footnotes

1 Observation of treatment patterns with LuCEntis and real-life ophthalmic monitoring, including optional OCT in Approved iNdications.

2 Cologne Image Reading Centre, University of Cologne; GRADE Reading Centre, University of Bonn; M3 Reading Centre, Eye Centre at St. Franziskus Hospital, Muenster.

## References

[1] Sacconi R et al (2019) Emerging therapies in the management of macular edema: a review [Version 1; peer review: 2 approved]. F1000Research 8(F1000 Faculty Rev):1413 10.12688/f1000research.19198.1 PMC6694451 31448093

[2] Hoerauf H et al (2016) Clinical efficacy and safety of ranibizumab versus dexamethasone for central retinal vein occlusion (COMRADE C): A European label study. Am J Ophthalmol 169:258–267. 10.1016/j.ajo.2016.04.020 27163237 10.1016/j.ajo.2016.04.020

[3] Heier JS et al (2012) Ranibizumab for macular edema due to retinal vein occlusions: long-term follow-up in the HORIZON trial. Ophthalmology 119(4):802–809. 10.1016/j.ophtha.2011.12.005 22301066 10.1016/j.ophtha.2011.12.005

[4] Tadayoni R et al (2017) Sustained benefits of ranibizumab with or without laser in branch retinal vein occlusion: 24-month results of the BRIGHTER study. Ophthalmology 124(12):1778–1787. 10.1016/j.ophtha.2017.06.027 28807635 10.1016/j.ophtha.2017.06.027

[5] Itoh Y et al (2016) Optical coherence tomography features in diabetic macular edema and the impact on Anti-VEGF response. Ophthalmic Surg Lasers Imaging Retina 47(10):908–913. 10.3928/23258160-20161004-03 27759856 10.3928/23258160-20161004-03 PMC5512288

[6] Bahrami B, Hong T, Gilles MC, Chang A (2017) Anti-VEGF therapy for diabetic eye diseases. Asia Pac J Ophthalmol 6(6):535–45. 10.22608/APO.2017350 10.22608/APO.2017350 29076303

[7] Wecker T et al (2017) Five-year visual acuity outcomes and injection patterns in patients with pro-re-nata treatments for AMD, DME, RVO and myopic CNV. Br J Ophthalmol 101(3):353–359. 10.1136/bjophthalmol-2016-308668 27215744 10.1136/bjophthalmol-2016-308668 PMC5339568

[8] Liakopoulos S et al (2020) ORCA study: real-world versus reading centre assessment of disease activity of neovascular age-related macular degeneration (nAMD). Br J Ophthalmol 104(11):1573–1578. 10.1136/bjophthalmol-2019-315717 32066561 10.1136/bjophthalmol-2019-315717 PMC7587226

[9] Schmidt-Erfurth U et al (2020) Application of Automated Quantification of Fluid Volumes to Anti-VEGF Therapy of Neovascular Age-Related Macular Degeneration. Ophthalmology 127(9):1211–1219. 10.1016/j.ophtha.2020.03.010 32327254 10.1016/j.ophtha.2020.03.010

[10] Müller S et al (2021) Questionnaire for the assessment of adherence barriers of intravitreal therapy: The ABQ-IVT. Int J Retina Vitreous 7(1):43. 10.1186/s40942-021-00311-x 34078475 10.1186/s40942-021-00311-x PMC8170736

[11] Heimes B et al (2016) Design of the ORCA module in the OCEAN study: Evaluation of SD-OCT results in daily routine practice. Ophthalmologe 113(7):570–580. 10.1007/s00347-016-0224-x 26868827 10.1007/s00347-016-0224-x

[12] Callizo J et al (2019) Real-world data: ranibizumab treatment for retinal vein occlusion in the OCEAN study. Clin Ophthalmol 13:2167–2179. 10.2147/OPTH.S209253 31806930 10.2147/OPTH.S209253 PMC6847987

[13] Ziemssen F et al (2017) Demographics of patients receiving Intravitreal anti-VEGF treatment in real-world practice: Healthcare research data versus randomized controlled trials. BMC Ophthalmol 17(1):7. 10.1186/s12886-017-0401-y 28103831 10.1186/s12886-017-0401-y PMC5244516

[14] Brinkmann CK et al (2019) Baseline diagnostics and initial treatment decision for anti-vascular endothelial growth factor treatment in retinal diseases : Comparison between results by study physician and reading centers (ORCA/OCEAN study). Ophthalmologe 116(8):753–765. 10.1007/s00347-018-0805-y 30367231 10.1007/s00347-018-0805-y

[15] der Stellungnahme DOG (2020) der RG und des BVA zur Therapie des diabetischen Makulaödems. Klin Monbl Augenheilkd 237(03):325–352. 10.1055/a-1097-3440 32182632 10.1055/a-1097-3440

[16] Berufsverband der Augenärzte Deutschlands e. V. (BVA), Deutsche Ophthalmologische Gesellschaft (DOG), Retinologische Gesellschaft e. V. (RG) (2018) Statement of the Professional Association of Ophthalmologists (BVA), the German Ophthalmological Society (DOG) and the Retinological Society (RG) on intravitreal treatment of vision-reducing macular edema by retinal vein occlusion : Treatment strategies, status 24 April 2018. Ophthalmologe 115(10):842–854. 10.1007/s00347-018-0775-0 30143857 10.1007/s00347-018-0775-0

[17] Schmidt-Erfurth U et al (2017) Guidelines for the management of diabetic macular Edema by the European society of Retina specialists (EURETINA). Ophthalmologica 237(4):185–222. 10.1159/000458539 28423385 10.1159/000458539

[18] Schmidt-Erfurth U et al (2019) Guidelines for the management of retinal vein occlusion by the European society of Retina specialists (EURETINA). Ophthalmologica 242(3):123–162. 10.1159/000502041 31412332 10.1159/000502041

[19] Stallworth JY et al (2020) Treatment patterns and clinical outcomes for central retinal vein occlusion in the antivascular endothelial growth factor era. J VitreoRetinal Dis 4(1):13–21. 10.1177/2474126419878922 10.1177/2474126419878922 PMC9976087 37009559

[20] Massin P et al (2019) Real-world outcomes with ranibizumab 0.5 mg in patients with visual impairment due to diabetic macular edema: 12-month results from the 36-month BOREAL-DME study. Ophthalmic Res 62(2):101–10. 10.1159/000497406 30928985 10.1159/000497406

[21] Ciulla TA et al (2018) Real-world outcomes of anti-vascular endothelial growth factor therapy in diabetic macular edema in the United States. Ophthalmol Retina 2(12):1179–1187. 10.1016/j.oret.2018.06.004 31047187 10.1016/j.oret.2018.06.004

[22] Ziemssen F et al (2018) intravitreal ranibizumab therapy for diabetic macular edema in routine practice: Two-year real-life data from a non-interventional. Multicenter Stud Germany Diabetes Ther 9(6):2271–2289. 10.1007/s13300-018-0513-2 10.1007/s13300-018-0513-2 PMC6250630 30288700

[23] Volkmann I et al (2020) Individualized treat-and-extend regime for optimization of real-world vision outcome and improved patients’ persistence. BMC Ophthalmol 20(1):122. 10.1186/s12886-020-01397-x 32228517 10.1186/s12886-020-01397-x PMC7104494

[24] Sivaprasad S et al (2016) Impact of injection therapy on retinal patients with diabetic macular edema or retinal vein occlusion. Clin Ophthalmol 10:939–946. 10.2147/OPTH.S100168 27307696 10.2147/OPTH.S100168 PMC4888735

[25] Ciulla TA et al (2021) Visual acuity outcomes and anti-VEGF therapy intensity in diabetic macular oedema: a real-world analysis of 28 658 patient eyes. Br J Ophthalmol 105(2):216–221. 10.1136/bjophthalmol-2020-315933 32265201 10.1136/bjophthalmol-2020-315933 PMC7848066

[26] Ciulla TA (2020) Visual acuity outcomes and anti-vascular endothelial growth factor therapy intensity in macular edema due to Retinal vein occlusion: A real world analysis of 12,214 Eyes. Invest Ophthalmol Vis Sci 61(7):3523

[27] Roberts PK et al (2020) Quantification of fluid resolution and visual acuity gain in patients with diabetic macular edema using deep learning: A post hoc analysis of a randomized clinical trial. JAMA Ophthalmol 138(9):945–953. 10.1001/jamaophthalmol.2020.2457 32722799 10.1001/jamaophthalmol.2020.2457 PMC7378869

[28] Vogl WD et al (2017) Analyzing and predicting visual acuity outcomes of Anti-VEGF therapy by a longitudinal mixed effects model of imaging and clinical data. Invest Ophthalmol Vis Sci 58(10):4173–4181. 10.1167/iovs.17-21878 28837729 10.1167/iovs.17-21878

[29] Berens P et al (2019) Proprietary data formats block health research. Nature 565(7740):429. 10.1038/d41586-019-00231-9 30675049 10.1038/d41586-019-00231-9

