# Supplementary material for: Interpretation of SD-OCT imaging data in real-life conditions versus standardized reading centre analysis in eyes with diabetic macular oedema or macular oedema secondary to retinal vein occlusion: 24-month follow-up of the ORCA study
Source: Graefes Arch Clin Exp Ophthalmol. 2024 Sep 19;263(1):131–9. doi: 10.1007/s00417-024-06579-7 (PMC11807050; doi:10.1007/s00417-024-06579-7)
Supplement: Supplementary file 1 — Supplementary file1 (PDF 488 KB) [file 417_2024_6579_MOESM1_ESM.pdf]

Online Resources

Title

Interpretation of SD-OCT imaging data in real-life conditions versus standardized reading centre analysis in eyes with diabetic macular oedema or macular oedema secondary to retinal vein occlusion: 24-month follow-up of the ORCA study

Journal

Graefe's Archive for Clinical and Experimental Ophthalmology

Author information

Georg Spital,[1] Steffen Schmitz-Valckenberg,[2,3] Bettina Müller,[4] Erika Liczenczias,[4] Petrus Chang[2], Britta Heimes-Bussmann,[1] Focke Ziemssen,[5,6] Sandra Liakopoulos,[7,8*] For the ORCA study group

Affiliations:

[1] M3 Reading Centre, Eye Centre at the St. Franziskus Hospital, Münster, Germany

[2] GRADE Reading Centre, Department of Ophthalmology, University of Bonn, Bonn, Germany

[3] John A. Moran Eye Centre, Department of Ophthalmology & Visual Sciences, Utah, Salt Lake City, UT, USA

[4] Novartis Pharma GmbH, Nuremberg, Germany

[5] Centre for Ophthalmology, Eberhard Karls University, Tübingen, Germany

[6] Department of Ophthalmology, Leipzig University Hospital, University of Leipzig, Germany

[7] Cologne Image Reading Centre, Department of Ophthalmology, Faculty of Medicine and University Hospital Cologne, Cologne, Germany

[8] Department of Ophthalmology, Goethe-University, Frankfurt, Germany

*Corresponding author

Sandra Liakopoulos, MD, Department of Ophthalmology, Goethe-University Frankfurt, Germany, sandra.liakopoulos@kgu.de

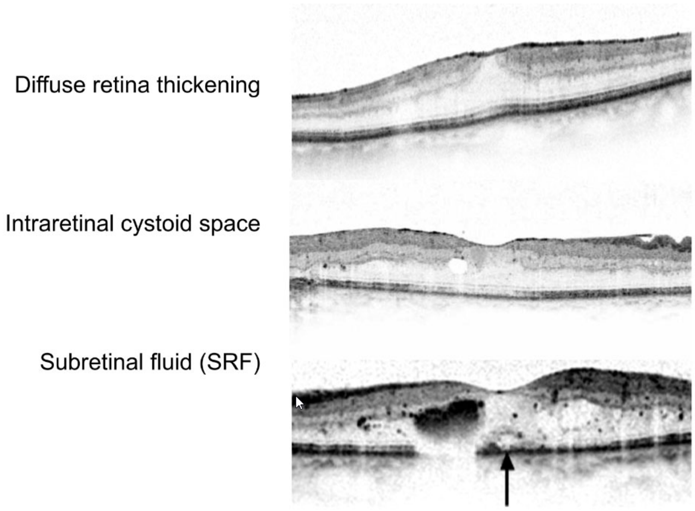

Online Resource 1

Example SD-OCT scans illustrating the minimal standard for clear identification of signs for “disease activity” in macular oedema. OCT signs of “disease activity” were defined as presence of intraretinal fluid [IRF] and/or subretinal fluid [SRF] with foveal involvement and/ or diffuse retinal thickening. SD-OCT, spectral domain optical coherence tomography.

Online Resource 2
Patients excluded due to conflicting physician and RC diagnoses

Physician diagnosis	RC diagnosis	N (patients)
DME	Macular telangiectasia	1
DME	Macular hole	1
DME	Macular dystrophy	1
DME	DR without ME	4
DME	CNV	1
DME	No ME, no DR	1
DME	No ME, subretinal scar	1
DME	nAMD	1
RVO	No ME, no RVO	2
RVO	nAMD	1

CNV, choroidal neovascularization; DME, diabetic macular oedema; DR, diabetic retinopathy; ME, macular oedema; RC, Reading Centre; RVO, retinal vein occlusion

Online Resource 3.

Demographic and clinical characteristics at baseline and during follow-up (grey).

Characteristics	ORCA DME cohort (N=77 patients)	ORCA RVO cohort (N=56 patients)
Age (years; mean [SD]))	67.4 [10.5]	71.6 [10.1]
Sex (patient; n, %)		
Male	38 (49.4%)	20 (35.7%)
Female	39 (50.7%)	36 (64.3%)
Treatment naïve (patient; n, %)	52 (67.5%)	38 (67.9%)
Baseline VA study eye (ETDRS letters; mean [SD])	63.7 [14.2]	56.1 [15.9]
Type of RC diagnosed RVO		
Central RVO (n, %)	N/A	16 (28.6%)
Branch RVO (n, %)	N/A	28 (50.0%)
Not assessable (n, %)	N/A	12 (21.4%)
Previous eye surgical or medical procedures (n, %)		
Laser pre-treatment#	25 (32.5%)	4 (7.1%)
Intraocular lens implant	4 (5.2%)	2 (3.6%))
Vitrectomy	1 (1.3%)	N/A
Ranibizumab injections during follow-up (mean, [SD])		
Months 1–6	4.05 [1.3]	3.66 [1.5]
Months 7–12	1.45 [1.8]	1.45 [1.7]
Months 13–18	1.14 [1.4]	1.11 [1.4]
Months 19–24	1.28 [1.6]	0.71 [1.1]
OCT scans per patient (mean, [SD])		
Months 1–6	4.6 [2.1]	4.4 [2.2]
Months 7–12	3.0 [2.2]	2.8 [2.3]
Months 13–18	2.7 [2.5]	2.2 [2.3]
Months 19–24	2.2 [2.2]	1.8 [2.0]

DME, diabetic macular oedema; ETDRS, Early Treatment Diabetic Retinopathy Study; N/A, not applicable; RVO, retinal vein occlusion; SD, standard deviation; VA, visual acuity

#Laser treatment more than 7 days before baseline BCVA examination.

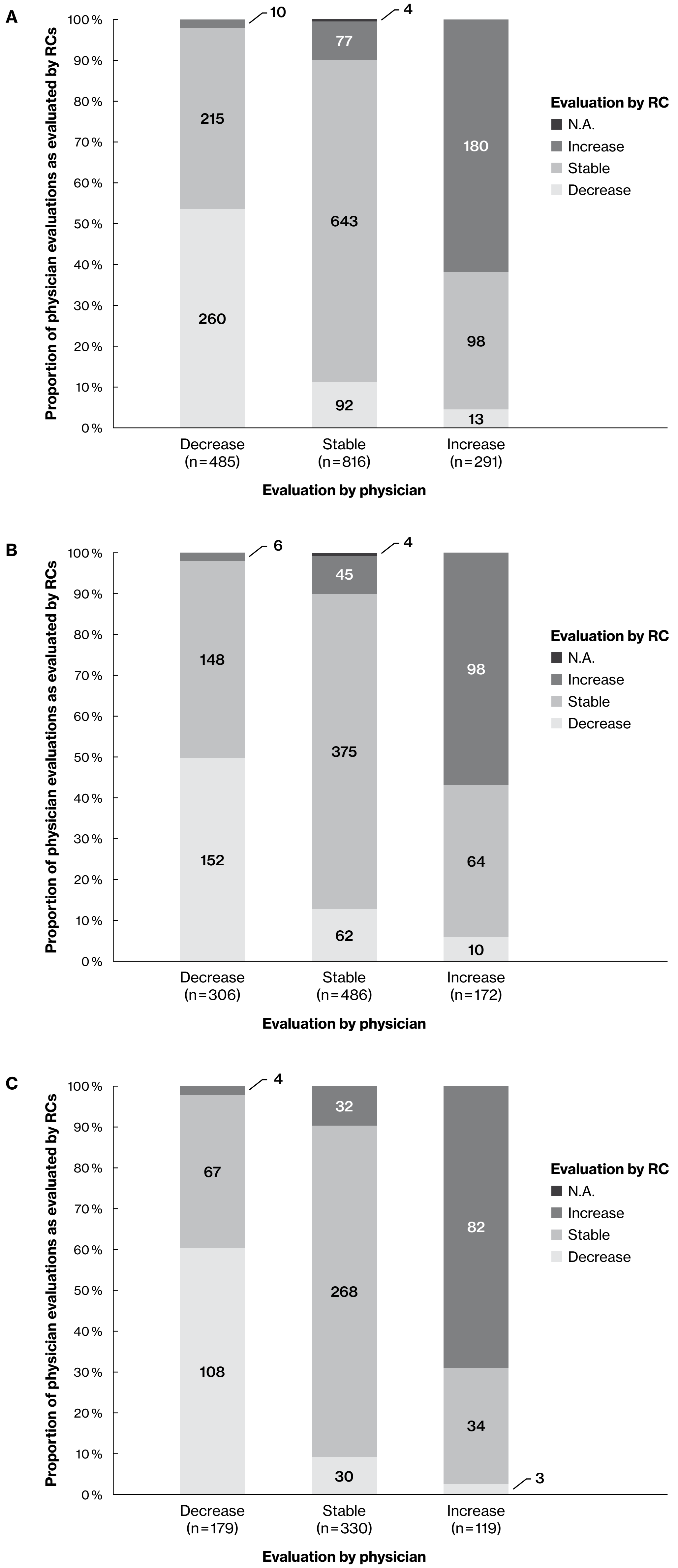

Online Resource 4

Physician and RC evaluation of change in the presence of IRF/SRF with foveal component in the combined ME cohort (A); ORCA-DME cohort (B); and ORCA-RVO cohort (C). DME, diabetic macular oedema; ME, macular oedema; RVO, retinal vein occlusion.

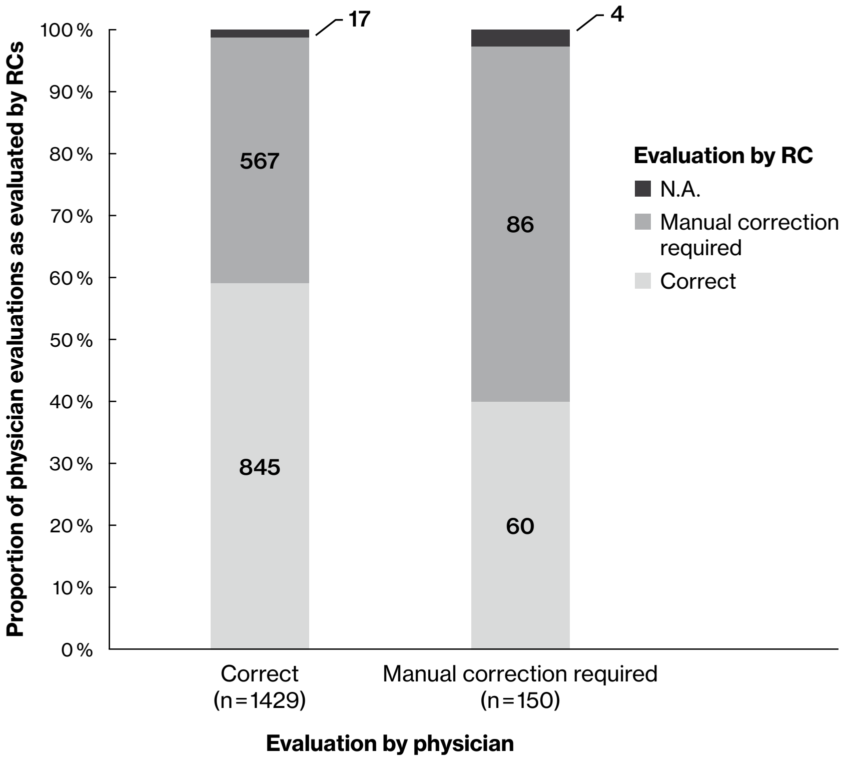

Online Resource 5

Comparison of assessment of grid positioning by physicians and RCs. N.A., Missing/Not assessable; RC, reading centre.

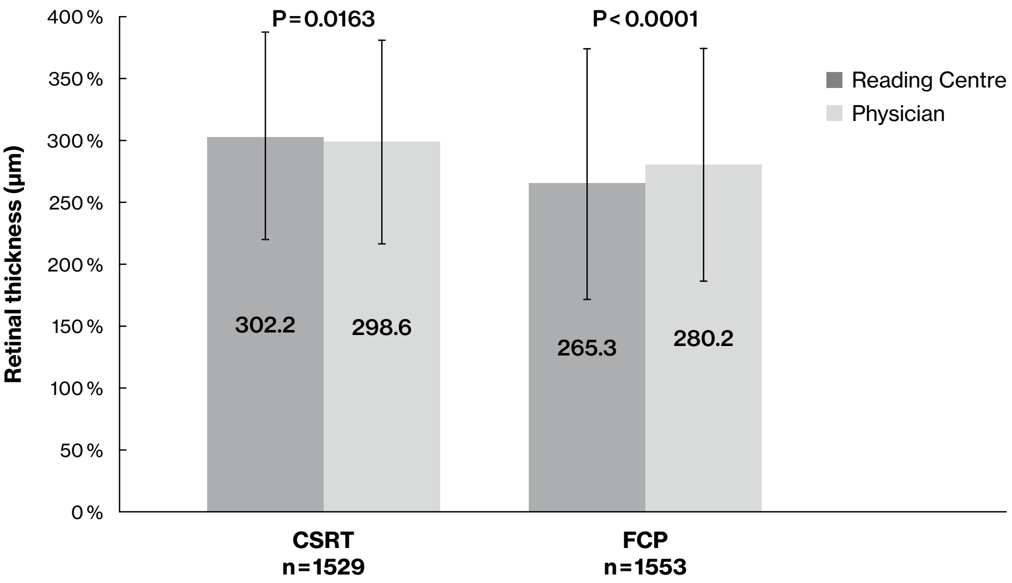

Online Resource 6

Central retinal thickness parameters (CSRT and FCP) as measured by RCs and physicians. Mean ± SD is shown for each parameter. CSRT, central subfield retinal thickness; FCP, foveal centre point; RC, reading centre. Note: Thickness in the central subfield was measured from internal limiting membrane to the photoreceptors and foveal centre point thickness was measured from the internal limiting membrane to the Bruch's membrane. In the DME cohort, physicians reported FCPT to be 291.0±71.5 µm whereas RCs reported 269.2 ±88.0 µm (mean difference: 23.4±60.3 µm; P<0.0001; n=946 scans). In the RVO cohort, FCPT measurements evaluated by physicians were 263.4±118.9 µm and RCs reported 259.2±134.1 µm (mean difference: 8.3±78.3 µm; P=0.009; n=607 scans).

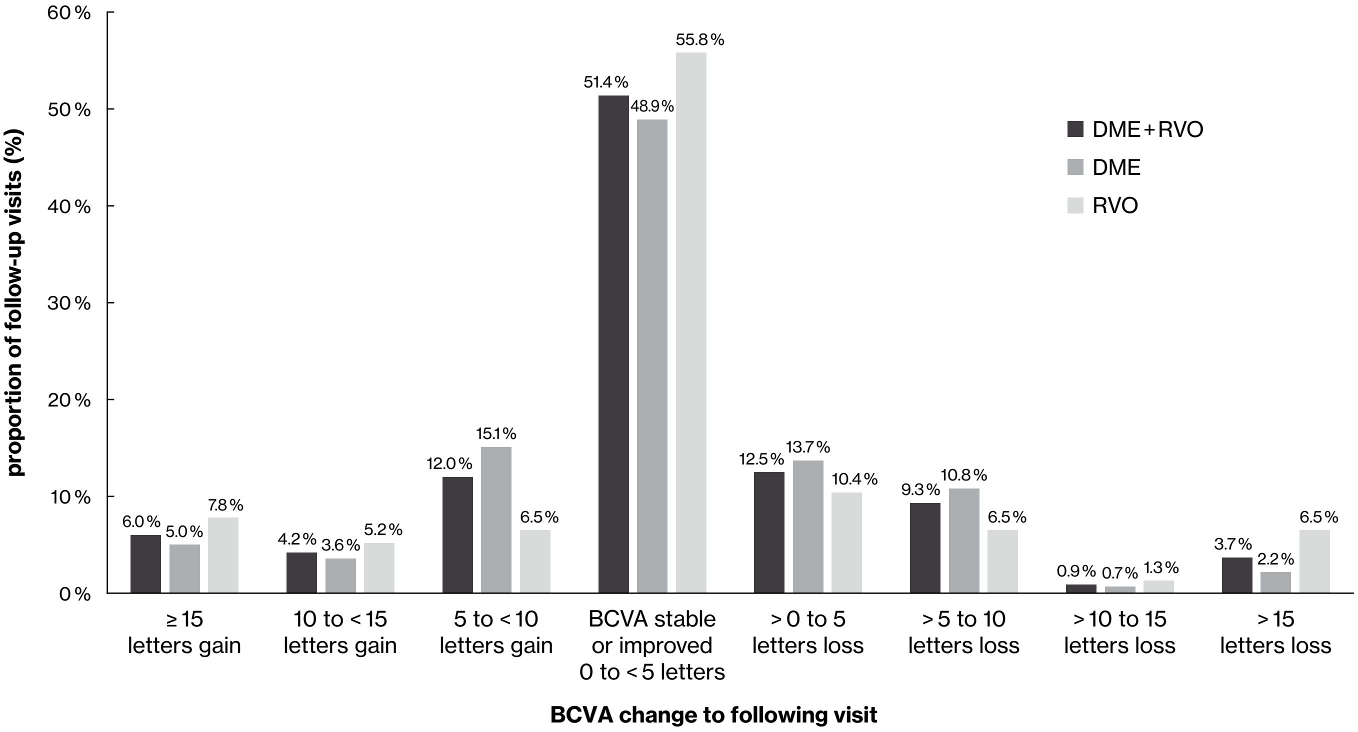

Online Resource 7

Changes in visual acuity from the visit when patients were not injected even though RCs detected signs of foveal IRF/SRF to the following visit. Cases, where RC analysis could justify a ‘watch and wait’ approach as comprehensive were excluded.

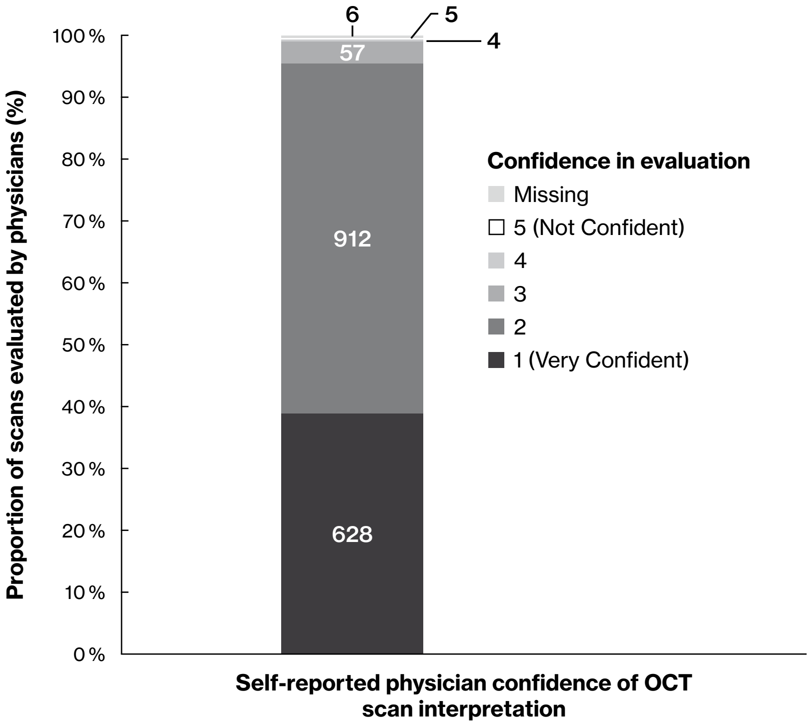

Online Resource 8
Physicians’ self-reported level of confidence at OCT scan interpretation ranked on a scale from 1 (very confident) to 5 (not confident). n = 1612 OCT scans. OCT, optical coherence tomography.

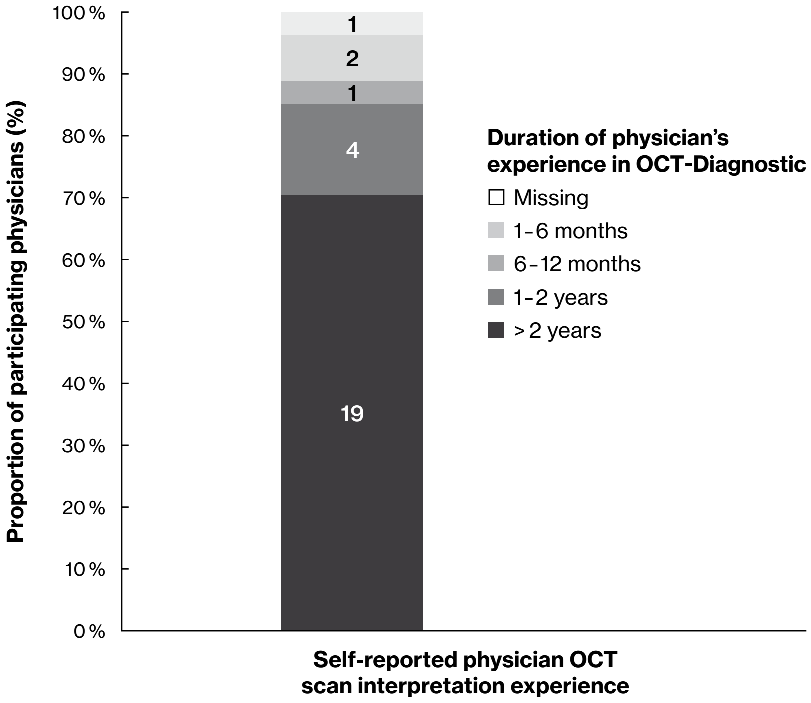

Online Resource 9

Physicians' self-reported experience in OCT scan interpretation. n = 27 physicians. OCT, optical coherence tomography.

Online Resource 10

Participants of the ORCA study group.

ORCA study group	
**Center (Hospital/Praxis)**	**Place**
Praxis Dr. Hälbig, Wolfgang	Augsburg
Praxis Dr. Brandt, Stefanus	Aurich
Gemeinschaftspraxis Dr. Ruf, Dr. Wille	Bad Gandersheim
Praxis Tyrtania, Thomas Dr.	Bad Kreuznach
Praxis Dr. Schmidt-Betschel, Wolfram	Bad Wildungen
Gemeinschaftspraxis Dr. König, Dr. Wißmann	Baden - Baden
Gemeinschaftspraxis Dr. Maximiliane Klatt, Dr. Alexander Klatt, Dr. Fabia Müller-Groh	Bassum
Augenzentrum Lichterfelde West Dr. Wiemer, Dr. Kaulen, Dr. Hänsgen, Dr. Galanski	Berlin
Gemeinschaftspraxis Dr. Gross, Bastienne Viehrig	Berlin
Gemeinschaftspraxis Dr. Pache, Dr. Planert, Dr. Engelhardt, Dr. Kube	Bielefeld
Augenklinik-Augenarztpraxis Schlosscarré Dr. Grohmann, Dr. Kamouna (Dr. Llacer)	Braunschweig
Praxis Dr. Grüb, Mathias	Breisach am Rhein
Krankenhaus St. Joseph-Stift GmbH (Oberarzt Dr. Cil)	Bremen
Gemeinschaftspraxis Dr. Berger, Dr. Niehuus	Bremerhaven
Praxis Dr. Lauritzen, Kyra	Buchholz
Praxis Dr. Brohr, Stephan	Burghausen
Gemeinschaftspraxis Dr. Stolzenberg, Dr. Haupeltshofer	Castrop-Rauxel
Praxis Dr. Niederstraßer, Dirk	Cuxhaven
Gemeinschaftspraxis Dres Steiner	Dannenberg
Klinikum Darmstadt GmbH (Prof. Emmerich)	Darmstadt
Praxis Krause, Roswitha Dr.	Dortmund
Gemeinschaftspraxis Dr. Müller-Holz , Dr. Riedel	Dresden
Universitätsklinikum Augenklinik Düsseldorf Dr. Guthoff, Rainer	Düsseldorf
IIO (Internationale Innovative Ophthalmochirugie) (Kaymak, Hakan)	Düsseldorf
Praxis Dr. Mohammadi, Babak	Düsseldorf
Augenklinik Flensburg Treumer, H. Prof. Dr. med. (Elshinnawi, Ahmed)	Flensburg
Praxis Dr. Brändle, Jürg	Füssen
Praxis Dr. Externbrink, Piet	Geseke
Praxis Fontana, Rainer Dr.	Grünstadt
Praxis Dr. Machnik, Bertram	Hamburg
Gemeinschaftspraxis Dr. Kaupke, Görges, Dr. Miebach	Hamburg
Praxis Dr. Wichmann, Wolfgang	Hannover
KRH Klinikum Nordstadt (Prof. Dr. Burkhard Wiechens)	Hannover
Praxis Dr. Pfeufer, Eva-Maria	Hildburghausen
Gemeinschaftspraxis Dr. Schmucker, Dr. Pusch	Hirschaid
Gemeinschaftspraxis Dr. Stephan Kilias, Urte Fetter, Dr. Ingmar Fetter	Hoppegarten
Praxis Dres. Maier und Liedtke-Maier	Hösbach
Praxis Dr. Grasbon, Thomas	Ingolstadt
Augen Allianz Zentrum Bayern Mitte (Dr. Sandra Oehmig)	Ingolstadt
Gemeinschaftspraxis Dr. Berens, Dr. Schumacher, Dr. Saschin	Karlsruhe
Augencentrum Koblenz (Dr. Berenstein)	Koblenz
St. Elisabeth-Krankenhaus Klinik für Augenheilkunde (Dr.Berk)	Köln
Augen + Laserzentrum (Dr. Kiraly)	Leipzig
Praxis Dr. David, Martina	Lohne
Praxis Dres. Burkhard Burkhard, Carsten Dr.	Lohne
Gemeinschaftspraxis Dr. Beckendorf, Dr. Kleineidam, Dr. Kruse	Lübeck
Universitätsklinikum LübeckKlinik für Augenheilkunde (Prof. Grisanti)	Lübeck
Gemeinschaftspraxis Dr. Asani und Kollegen (Fr. Kjato, Azra)	Lünen
Gemeinschaftspraxis Prof. Dr. Neuhann (Dr. Bornhauser)	München
Praxis Dr. Socrates Dimitriou (Dr. Schultmeyer)	München
Augenklinik Airport München (Bechmann, Martin)	München-Flughafen
Westfälische-Wilhelms-Universität Münster (Prof. Eter)	Münster
Praxis Tesnau, Renata Dr.	Mutterstadt
Praxis Dr. Wildner, Bodo	Neustadt
Praxis Dr. Wildner, Annette	Neustadt
Praxis Dr. Winkler von Mohrenfels, Christoph	Neutraubling
Praxis Dr. Gockeln, Roland	Nienburg
Gemeinschaftspraxis Dr. Lanzrath, Dr. Schlese, Dr. Breuer	Oldenburg
Praxis Lenz, Christof Dr.	Paderborn
Praxis Dr. Garus, Lena	Pfaffenhofen
Gemeinschaftspraxis Dr. Kallmann, Fanihagh & Partner (Fanihagh)	Ratingen
Gemeinschaftspraxis Dr. Kallmann, Fanihagh & Partner (Dr. Kallmann)	Ratingen
Gemeinschaftspraxis Dr. Claus Fuchs, Dr. Barbara Fuchs-Koelwel	Regensburg
Praxis Dr. Scheffler, Martin	Rhauderfehn
Augenarztpraxis Rostock Mitte	Rostock
Praxis Kirchhoff, Ulrike Dr.	Rotenburg
Belenus Augenzentrum, Gemeinschaftspraxis Dres. Frank, Fuchs, Briesen	Siegen

Praxis Dipl. Med. Landmann, Heike	Stendal
Praxis Dr. Czechowski, Edelgard	Stendal
Praxis Dr. Kaiser, Adam	Stuttgart
Praxis Grote-Schmidt, Annette Dr.	Tönisvorst
Universitätsaugenklinik Tübingen (Prof. Dr. Ziemssen)	Tübingen
Praxis Dr. Hufenbach, Ulrich	Wernigerode
Gemeinschaftspraxis Timm, A., Dr. Beyer, Marion	Wismar
Praxis Steinbach, Claus-Michael Dr.	Wuppertal
Praxis Murovski, Simo Dr. (Univ. Skopje)	Zschopau
